# Efficient qualitative risk assessment of pipelines using relative risk score based on machine learning

**DOI:** 10.1038/s41598-023-38950-9

**Published:** 2023-09-10

**Authors:** C. N. Vanitha, Sathishkumar Veerappampalayam Easwaramoorthy, S. A. Krishna, Jaehyuk Cho

**Affiliations:** 1 Department of Computer Science and Engineering, Kongu Engineering College, Erode, India; 2 Department of Software Engineering, Jeonbuk National University, Jeonju-si, Jeollabuk-do Republic of Korea; 3 Department of Mechatronics Engineering, Kongu Engineering College, Erode, India

**Keywords:** Engineering, Mathematics and computing

## Abstract

Pipelines are observed one of the economic modes of transport for transporting oil, gas, and water between various locations. Most of the countries in the world transport petroleum and other flammable products through underground pipelines. The underground and aboveground pipelines are facing various damages due to corrosion, dents, and ruptures due to the environment and operational fluid conditions. The danger of leaks and accidents increases as a result of these damages. Pipelines must be evaluated on a regular basis to make sure they are fit for transmission. By evaluating the effects of damages and the possibility of catastrophic failures using a variety of techniques, pipeline integrity is controlled. Applying the relative risk scoring (RRS) technique, pipeline failures are predicted. One of the probabilistic techniques used to forecast risk based on an impartial assessment is machine learning. With different parameters like corrosion, leakage, materials, atmosphere, surface, earth-movements, above-ground and underground facilities, etc., the RRS method provides an accuracy of 97.5% in identifying the risk and gives a precise classification of risk, whether the pipeline has a high, medium, or low risk without any delay on the prediction compared with Naive Bayes, decision tree, support vector machine, and graph convolutional network.

## Introduction

India's economy is growing rapidly, necessitating increased hydrocarbon transport capacity. An item of machinery called a pipeline is made to move material constantly or irregularly from one place to another. Modern technologies prefer pipelines over other modes of transportation due to financial and safety reasons. Pressure is applied as highly flammable hydrocarbon material is transported across pipelines across the nation, frequently close to dense populations and places with a high environmental consciousness. Allocating a lot of money to preventive measures activities for mitigation and detection will help you analyse the risk presented by international pipelines effectively. To protect individuals, the general public, the environment, and property, more safety must be provided. An effective algorithm is required to simplify the processes and lower the failure rate of gas pipelines^1^.

Pipelines pose risks such as jet fire, unconfined vapour cloud explosion, flash fire, delay ignition, pool fire, and toxicity. There are many integrity management tools, like cathodic protection, inline inspection, hydro testing, surveillance, direct assessment and evaluations, pipeline equipment health monitoring, and thickness assessment^2,3^. The pipeline threats are categorised as fixed, non-stationary, and stationary. The non-stationary threats are outer erosion, inner erosion, and strain erosion splitting. Pipeline defects can occur during the manufacturing process, as well as during welding or fabrication^4^. Some of the failures that occur as a result of gasket toxicity (joint failure, guide apparatus malfunction, and clinch force out failure) must also be considered when considering the threat to stable equipment.

Many other threats should be considered as time-independent threats while analysing the threats in equipment and external factors. As with computer hackers, some third parties may damage pipeline equipment or perform incorrect operations, causing the pipeline to malfunction. Natural disasters^5^ like lightning, heavy rains, floods, weather-related events, and earth movements also affect the quality of pipelines. Due to the severity of the composite risk from all threats, pipeline sections may be prioritised for integrity assessment.

The overall risk value for a specific pipeline section is determined by the likelihood of failure and its consequences, taking into account all relevant dangers. Risk ranking^6^ will be determined for pipeline segments found to be at high risk in order to arrange the integrity evaluation. Prior to the execution of any pipeline framework advancements, an exact cycle will be put in place to make sure that impending changes are evaluated for their potential risk effects on the pipeline. The RRS method concentrates on using the relative risk scoring method of machine learning to perform the integrity and qualitative risk assessment of pipelines while taking into account all of these situations and factors.

The focal objectives of this research are:

1. There are other studies that are dependent on specific aspects, such as leakage, corrosion, etc., but none of them offer a specific solution for all of the problems that are widespread.
2. The Research focus to assess the risk of pipelines by calculating the Relative Risk Score (RRS) method.
3. The RRS approach takes many parameters into account that could impact a pipeline. The methodology that is being suggested here aims to lessen the harm happens due to harmful gases, chemicals, and petroleum products inflict on people and other living things.
4. To demonstrate the superior effectiveness of the novel approach the RRS method contrast it with Naive Bayes, Decision tree, SVM, and GCN.

The arrangement of this research paper is as per the following: The existing methodologies and literature background for pipeline assessment are presented in Segment 2. The terminologies related to the pipeline assessment are explained in Segment 3. The proposed RRS algorithm using machine learning is elaborated in Segment 4. The experimental setup and the discussion of results are presented in segment 5, and the research is concluded in segment 6.

## Literature review

### Risk assessment of chemical pipelines

The use of machine learning techniques in pipeline risk assessment has been a topic of increasing interest in recent years. Sohaib et al.^5^ proposed a method for detecting leaks in circular water storage tanks in the chemical sector using acoustic emissions. Support vector machines are used to locate the exact location of the crack or leakage. Mazumder et al.^7^ used machine learning algorithms to analyze the risk of failure of a steel pipeline. The research developed an alternative to statistically intensive analytical methods to estimate the steel pipeline failure threat. However, the research could not be fully realized due to the lack of adequate data for use in actual situations.

Yang et al.^8^ demonstrated urban gas data-driven pipeline accidents and consequences assessment using machine learning. The accidents in UPN may cause environmental disasters. Urban pipeline maintenance is related to the major facets of data. The work uses conventional assessment for risk models like the Kent index method and analytic evaluation indicators. The graph convolutional network (GCN) technique is used to assess the risk associated with pipelines. Liu and Bao^9^ reviewed automated conditions for the assessment of pipelines with machine learning. Pipelines, which transport intense substances, play a significant role in societal safety and commercial prosperity. Pipeline condition assessments are required to identify the risk.

Wu et al.^10^ presented FTAP: A feature-transferring autonomous machine learning pipeline. Successful machine learning^11^ frequently involves significant involvement with algorithms and expert knowledge in the field. The ML methods heavily rely on domain-specific information. FTAP improves efficiency and performance. It has also achieved success in distance domain transfer learning. He et al.^12^ used Geographical Information System at the threat location to develop a genetic and back propagation prototype to forecast the occurrence of a geographical calamity and avert pipeline damage. The GA-BP model is used to estimate weights of the indication factor by utilising the benefits of analysing data and predictive analytics, which avoid the subjective authority of earlier calculation methods.

Malinowska et al.^13^ showed off a model that uses Mamdani fuzzy inference for the study. In this method, the hazards in the pipeline due to the estimated horizontal strains, the solidity of the material, the time span of an unfavourable effect, and the importance of the targets are evaluated on the basis of one level. The prototype is used to assess the risk of an arranged gas pipe with systematic variables. This can also be used to stimulate further scientific variations of the examined item. Bu et al.^14^ investigated how soil-atmosphere coupling affects gas pipeline^15^ leakage. The joining process of methane leakage was calculated using arithmetic stimulation, and the effects of many factors on methane convergence in FDZ and SDZ were investigated. This analysis provides guidelines for the planning of gas pipes, improving the installation of detectors, and gas leakage^16^ maintenance.

Liu et al.^17^ demonstrated a dynamic danger estimation prototype depending on system dynamics (SD). Most of the pipeline risk assessments provide a static outline of the process. This model is to manage both the issue of given activity and changes there within a period. The solution provided the dynamic developments of principles of buried pipelines threat are compatible with real circumstances. This prototype adequately distinguishes the temporal and spatial principles of threat evolution.

Adumene et al.^18^ presented a method that combines the semi-empirical corrosion prototype with material used and parametric uncertainties. The pipe failure rises with a rise in factor of difference at the minimum limit of futile, while reduces in the maximum limit of futile. This method provides an organized structure for choosing material and threat-based integrity management plans for sea pipelines.

Froeling et al.^19^ demonstrated the danger of dangerous hydrogen jet fire transported through gas pipelines^20^,^21^. This analysis focuses on threats associated with a dangerous hydrogen fire, such as an invisible spray with a gas pipeline^22^. Using analytical software, it performs a detailed fire imitation and condition of the artwork. When compared to a gas pipeline, the ignition effects influence the threat for reducing pipe diameters and causing hydrogen transference to capitulate an increased hazard. Taleb-Berroune et al.^23^ suggested a prototype for the risk assessment for the deterioration of pipelines utilising adaptive bow-tie (ABT) analysis. The ABT model used for this analysis is engrossed in microbiologically impacted pipeline deterioration, as well as a corrosion economic risk profile. This prototype serves as a procedure to recognize, evaluate, and maintain the corrosion of the pipeline.

Wang et al.^24^ initiated a susceptibility assessment technique for the danger assessment of the gas pipe system. It integrates the features of threat assessment and susceptible analysis methods. Risk susceptibility classifies its critical components into three categories: the operating status of the pipeline, transmission performance, and network features. A utility proposition is employed to identify the depth of the outturn. This method balances the lower part of the threat, trustability, and susceptibility curves to adequately recognise the unfavourable joints and pipelines damaging gas supply in a pipeline network.

Zulkifli and Salleh^25^ analysed the effect of movement and pipe construction actions at various pressures and pipe widths on the pipeline in the UTHM biodiesel plant. The Computational Fluid Dynamics (CFD) technique helped them to examine the velocity and pressure dispersion, while the Interaction of Fluid Structure (FSI) method enabled them to examine the stress distribution on the pipes according to their thicknesses. The study found that the breadth of the pipe affects the flow rate and pressure in the region of the pipe, and pressure diffusion is reduced as quality improves. Additionally, when pressure increases, the storm created within the pipe also increases.

### Risk assessment of buried pipelines

Li et al.^26^ proposed a method to analyse and maintain gas pipelines externally based on Bayesian networks. Buried gas pipelines^27^ are often exposed to natural calamities, leading to corrosion. To identify the cause of pipeline deterioration, the study used a defective tree prototype and implanted the maintenance plan in the Bayesian network. This approach provides an adequate maintenance plan for pipelines and reduces losses caused by external corrosion.

Jabbari et al.^28^ used a down-covered logical hierarchy procedure to assess the risk of poisonous gas discharge and outburst in gas pipelines^29^. An analysis of danger was carried out using MATLAB software, and the mass of each item in basic risk (BRI) was outlined in a reference frame. The study found that five-state norms determine the level of threat. This method is applicable to the security chief when making decisions associated with the hazardous evaluation of a gas pipeline.

Yin et al.^30^ presented an upgraded quantifiable danger assessment for gas pipelines, considering high-importance areas. Given that most gas discharge incidents in China occur in crowded areas, the study established two models: a failure probability^31^ model and a risk consequence model. This approach can adequately recognize significant areas and produce reliable outcomes.

Chen et al.^32^ proposed a danger analysis method for buried pipes based on an upgraded cloud variable weight thesis. The study offered a new way of using the cloud variable weight hypothesis to examine the hazard amount and consequential danger factor of a pipeline by setting up a threat evaluation index system for the pipeline. This method assists the supervisor in determining the threat level and sore point in the pipeline.

Wang et al.^33^ developed a technique for evaluating the existence of deteriorated pipeline balances and assessing the threat of pipeline defaulter conditions in the face of an unexpected current attack. The study used a Monte Carlo simulation approach to determine the impedance caused by electrochemical reactions and the unreliability of prototype variables. The balance course of the corrosive ecological variables remains remarkably constant, except for the dynamic stray current immersed by the pipeline.

Mederios et al.^34^ presented a technique to deal with multi-dimensional risks occurring in gas pipelines based on unexpected utility. The study accomplished multi-dimensional risk evaluation of gas pipelines in decision-making and used a non-expected utility method in the MRDU prototype. The deflection of usefulness was surveyed, and this included the benefit from an RDU (rank dependent utility)-based danger proposal. The study conducted similar outcome analysis and sensitivity testing, and this method provides great support to the decision-makers with regard to natural gas pipeline sections.

Ullah et al.^35^ assessed the rockburst patterns s of the Jinping-II hydropower project in China to enhance employee security in mining and geotechnical works. In the present work, the following methods were used to predict short-term rock burst threat: t-distributed stochastic neighbour embedding (t-SNE), K-means clustering, and extreme gradient boosting (XGBoost). The implied model's results provide an excellent standard to guide future short-term rock burst levels forecasting with excellent precision.

Kamran et al.^36^ proposed a model to reducing rockburst-related mortality. In this study, firstly,isometric mapping (ISOMAP) algorithm is used.then, ISOMAP was categorized using the fuzzy c-means algorithm (FCM) and at last, in order to predict different levels of the short-term rockburst dataset, KNearest Neighbour (KNN) was used. In the experiment's dataset, the suggested model properly categorised 96% of the rockburst occurrences.

Kamran et al.^37^ utilized the algorithms like Catboost and light gradient boosting machine (LightGBM) techniques with the objective to reduce the number of casualties and property damage associated with deep underground engineering tasks. Here, Catboost and light gradient boosting machine (LightGBM) techniques to examine several intriguing elements of mine fire statistics. The results show that LightGBM algorithms, having an accuracy of 92% and 89%, respectively, outperformed Catboost in terms of performance.

Kumaran et al.^38^ introduces a novel approach to foresee the stability of underground coal pillars utilising integrated unsupervised and supervised learning to roughly simulate the complicated behaviour of coal pillars. Kumaran et al.^39^ proposed a novel approach to forecast mine fire levels using a variety of machine learning approaches.

After examining various literature, pipelines are exposed to various physical and chemical environmental issues. These result in the explosion of chemicals, which lead to the spread of various diseases to humans, fire accidents, and natural calamities like land pollution, landslides, earthquakes, etc. The literature that is examined only with minimal parameters that affect the pipeline and does not contain important parameters to which the pipeline is exposed. In a specific assessment, the need for integrity and qualitative risk assessment of pipelines with various parameters such as corrosion, leakage, coating, materials, atmosphere, surface, earth movements, population, above-ground and underground facilities, and so on is identified. The existing methodologies focused only on the risk of pipelines and assessing that particular risk. This may lead to catastrophic disasters where the pipeline is being implanted. So, an efficient relative risk score (RRS) method is proposed by assessing the pipeline using the RRS method with various parameters that affect the pipelines, which is greatly helpful and safe to transmit materials through the pipelines.

## Related terminologies

### Pipeline integrity management (PIM)

Pipelines are the best-grounded and cheapest mode for transporting oil, fluids, and natural gas. Pipeline networks^2^ are large and complicated; they consume time and are often intensive for inquiring about a lot of pipelines. PIM is executed to reduce the possibility of remissness caused by the debasement and to maintain the programmability and security of pipelines. Despite the warnings to avoid transporting natural gas, fluids, and oil, these pipes are subjected to deteriorating conditions. The PIM are used to define the systematic approach, identify the possible risk by way of danger, and apply the preventive methods.

The research focuses on the basic objectives of pipeline integrity management (PIM) to improve confidence among the public in pipeline safety and operator management. It improves the operational processes to maintain the integrity of the pipeline. The primary goal of PIM is to ensure pipeline reliability, prevent incidents, and maintain the operation license. These integration activities generate the data with 3 V’s (a huge amount of volume, velocity, and variety) based on the pipeline’s length and the sensors and tools that are used to access the condition of pipelines. The pipeline should ensure not only the mechanical condition but also the operator's reliable operation, delivery duty, image, and estimation.

### Risk assessment

Danger estimation and management is the term used to describe the general method for identifying the danger and the risk factors that have the potential to cause damage. Analyzing and evaluating the problem that is associated with the hazard. The tools used for risk assessment are the risk matrix, decision tree, failure modes and effects analysis. Since the oil, fluids, and natural gas pipes are burnable, they will be dangerous and toxic. The outflow in the pipeline can cause catastrophic effects like fire explosions and environmental pollution. Risk assessments used to reduce risk include baseline, issue-based, and continuous risk assessments.

Pipeline assessment entails closely inspecting pipeline inner and outer sections to determine corrosion rates, flow modeling, and profile calculation. The two components of pipeline risks are leaks and ruptures. The risk in the pipeline^40^ can be monitored by using supervisory control and data acquisition systems, which collects data about the pipeline operations and transmit the data to engineers or technicians if some problem occurs. The security cameras and sensors, fibre-optic cables, and temperature sensors placed on or near the pipelines can also check for leakages and corrosion to prevent them from becoming dangerous.

### Machine learning in PIM

Machine learning^41^ concentrates on integrity, maintenance, inspection, analysing the crack, and preventing corrosion in pipelines. In pipeline integrity management, the two categories of ML classification and regression are used. Classification is used for detecting leakage, identifying the defect type, and predicting the level of risk in pipelines. Regression is used for calculating the size of the defect and predicting the rate of debasement in pipelines. For pipeline integrity management, clustering identical pipeline^42^ segments based on identical operating conditions, materials used for establishment, and debasement mechanisms are taken into account for the assessment of risk.

### Classifier techniques used in prediction

The Classification algorithm is a Supervised Learning technique utilized in order to categorize new observations, on the premise of training data. In classification, a system makes use of the dataset or observations that are provided to learn how to categorize fresh observations into various classes or groups. In this research, three classifier models such as support vector machine (SVM), Decision Tree and Naïve Bayes are used to identify the chance of pipeline failures, based on a variety of input characteristics.

#### Support vector machine (SVM)

Collecting and pre-processing the data is the initial stage in the use of SVM for pipeline risk assessment. Data collection on pipeline parameters, environmental variables, and information on previous pipeline failures are necessary.

1 $$y\_i(\left(w*x\_i\right)+b)=1\,for\,all\,i$$

where y_i is the class for the i-th data point, x_i is the input vector for the i-th data point, b is the bias term.

#### Decision tree

It functions by creating a tree-like model of choices and potential outcomes. Each node in a decision tree indicates a choice made in response to a particular characteristic or attribute of the data. Up until a stopping requirement is satisfied, the data are recursively divided into subsets based on the values of the characteristics. This terminating criterion can be a predetermined tree depth or the minimal quantity of samples needed in a leaf node. The leaf nodes represent the class labels. By estimating the possibility of a pipeline failure or leak based on different characteristics like the age of the pipeline, the material it is constructed of, the operating pressure, and the location, decision trees can be utilized for pipeline assessment.

2 $$Information\,Gain=Entropy\left(S\right)- [\left(Weighted\,Avg\right)*\left(Entropy\left(each\,feature\right)\right)]$$

The above Eq. 2 calculates how much information a feature provides us about a class

3 $$Entropy\left( S \right) = - P\left( {\text{positive classes}} \right) log_{2} P\left( {\text{positive classes}} \right) - P\left( {\text{negative classes}} \right) log_{2} P\left( {\text{negative classes}} \right)$$

4 $$gini\,index= 1- {\sum}_{j=1}^{C}{{\mathrm{P}}_{j}}^{2}$$

The above Eqs. 2, 3 and 4 calculate entropy and gini index. C also represents the number of events (groups). The entropy and Gini index is a measure of impurity or purity used while creating a decision tree.

#### Naïve Bayes

This is also using the different characteristics of pipeline to assess. It is possible to identify high-risk pipelines and set priorities for maintenance and inspection work by utilising a Naive Bayes algorithm for pipeline assessment. The Naive Bayes algorithm is excellent for huge datasets since it is easy to use and computationally effective. The Naive Bayes algorithm for pipeline evaluation involves utilising the Bayes theorem to determine the likelihood of a pipeline failure or leak given its characteristics.

## Proposed RRS methodology

- System model of risk assessment of pipelines using machine learning

The research focuses on qualitative risk assessment and the integrity of pipelines using relative risk scoring methods in machine learning. Relative risk scoring is an index model in which important conditions and activities of the pipeline are assigned numerical values (scores) that contribute to risk. Depending on the needs of the assessment, multiple layers of the layered hierarchy in which the relative risk scoring algorithm is designed may be necessary. Failure or consequence factors are investigated. The relative contribution to the risk, consequence, or total risk will determine the best course of action for risk minimization.

The relative risk score (ℜ) is calculated by sum of the Index Sum (µ) divided by the Leak Impact Factor.5

Index sum (µ) is the sum of Third-party index (Ɲ), Corrosion Index (ʩ), Design Index (ϑ) and the Incorrect Operation (Ѵ).

I. Third party index

The Third-Party Index (Ɲ) is the summation of the seven factors.6where X_1_ is the minimum depth cover. In this case, X1 is obtained by dividing the no. of inches covered by three [X1 = A1/3], where A1 is the number of inches covered. X_2_ is the activity level, X_3_ is the facilities in aboveground, X_4_ is the line locating, X_5_ is the public education, X_6_ is the Right of the way Condition, X_7_ is the patrol and n is the number of factors in third party index.

II. Corrosion Index

Corrosion Index (ʩ) is the summation of Atmospheric Corrosion (À), Internal Corrosion (ƛ), and Subsurface Corrosion (₰). Where À is the Atmospheric corrosion, ƛ is the internal corrosion and ₰ is the subsurface corrosion. Atmospheric corrosion (À) is calculated with the summation of three factors.

7 $$\grave{A} ={\sum }_{n=1}^{3}{Y}_{n}$$

where Y_1_ is the atmospheric exposure, Y_2_ is the atmospheric type and Y_3_ is the atmospheric coating.

Internal corrosion (ƛ) is calculated with the summation of corrosion in the product (ɥ) and internal production ($\gamma$).8 Subsurface corrosion (₰) is the summation of subsurface environment (*ψ*), Cathodic protection (ɰ) and coating ( ).

Subsurface environment (*ψ*) is the summation of corrosion in soil (ϒ) and mechanical corrosion (П).

9 $$\Psi = \Upsilon + \Pi$$

Cathodic protection (ɰ) is the summation of effectiveness () and interference potential ().
10

Coating ( ) is the summation of fitness (Ʊ) and condition (ȡ). 
11

From Eqs. (9), (10) and (11),12

From Eqs. (6), (7) and (12), 13 where ʩ is the corrosion index.

III. Design Index

Design index (ϑ) is the summation of safe factor (D_1_), fatigue (D_2_), surge potential (D_3_), verification of integrity (D_4_), land movements (D_5_).

14 $$\vartheta = \sum\limits_{(n = 1)}^{5} {D_{n} }$$

where ϑ is the Design index, n is the number of factors, D_1_ is the safe factor, D_2_ is the fatigue, D_3_ is the surge potential, D_4_ is the verification of integrity, D_5_ is the land movements.

IV. In-correct Operation Index

The In-correct Operation Index (Ѵ) is the sum of design (ɠ), construction (ɓ), operation (τ), and maintenance (ω). 15 where ɠ denotes the design, n denotes the number factors, ${M}_{1}$ is the Hazard identification, ${M}_{2}$ is the MAOP potential, ${M}_{3}$ is the safety system, ${M}_{4}$ is the material selection and ${M}_{5}$ is the checks. 16 where ɓ denotes the construction, n denotes the number factors, ${N}_{1}$ is the inspection value, ${N}_{2}$ is the Materials rating, ${N}_{3}$ is the joining value, ${N}_{4}$ is the backfills, ${N}_{5}$ is the handling and ${N}_{6}$ is the coating of pipelines.

17 $$\uptau ={\sum }_{n=1}^{7}{O}_{n}$$

where τ denotes the operation, n denotes the number factors, ${O}_{1}$ is the procedure, ${O}_{2}$ is communication test, ${O}_{3}$ is drug testing value, ${O}_{4}$ is the safety program, ${O}_{5}$ is the survey/maps/record, ${O}_{6}$ is the training and ${O}_{7}$ is the mechanical error preventer.

18 $$\upomega ={\sum }_{n=1}^{3}{P}_{n}$$

where ω denotes the Maintenance, n denotes the number factors, ${P}_{1}$ is the documentation, ${P}_{2}$ is the schedule and ${P}_{3}$ is the procedure.

From above Eqs. (15), (16), (17) and (18), 19

From Eqs. (6), (13), (14) and (19). 20

I. Product hazard

Product hazard is calculated by the summation of acute hazard and chronic hazard. 21 where δ denotes the acute hazard, η is the reactivity, ƕ is the flammability and ؏ is the toxicity.

22 $$\zeta ({\text{chronic}}\,{\text{hazard}}).$$

From the above (21) and (22), 23 where  denotes the Product hazard, δ is the acute hazard, ζ is the chronic hazard.

II. Leak volume

Leak volume (Final spill score) = (effective score of spill size) × (adjustment factor larger openings). 24 where £ denotes the Leak volume, ξ is the score of the spill size and is the Adjustment factor larger openings.

III. Dispersion

Dispersion is calculated by Operating pressure divided by 100. 25 where Ә denotes the dispersion and  denotes Operating pressure.

IV. Receptors

Receptor (ɮ) is the summation of population density (ρ), environment considerations () and high value areas (*ℏ*). 
26 where ɮ denotes Receptors, ρ is the population density,  is the Environmental considerations and *ℏ* is the High value areas.

From Eqs. (23), (24), (25) and (26), 27 where  denotes the Product hazard, £ is the Leak volume, Ә is the Dispersion and ɮ is the Receptors.

b. Architecture and work flow of risk assessment of pipelines using machine learning

**Figure 1:**
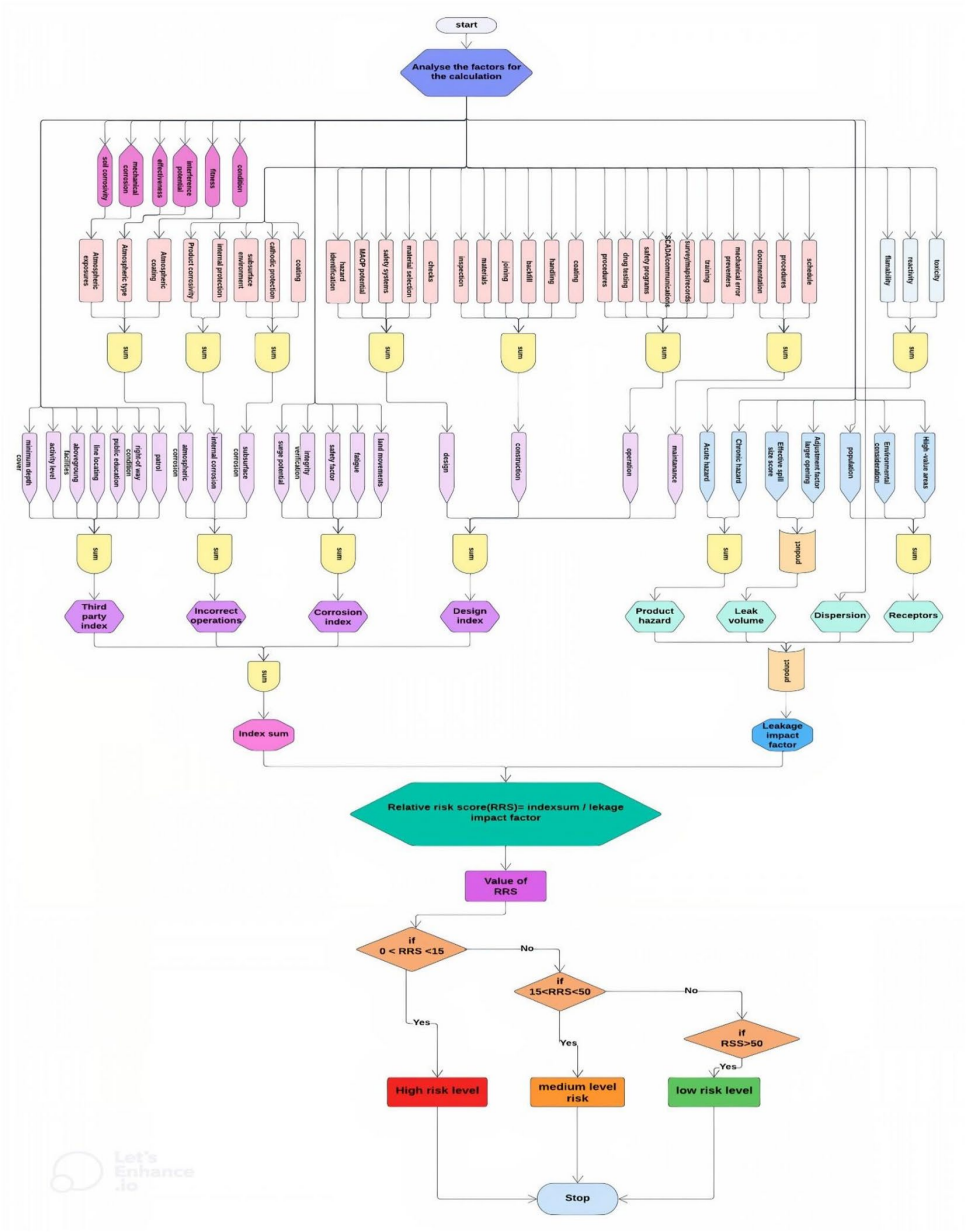
Flow chart for RRS method.

Figure 1 presents the complete flow of the system. The system first analyses various parameters that are needed to calculate the risk score of pipelines. Using that the Third-party index, corrosion index, Design index are calculated. Finally, with the index sum and the leakage impact factor the risk level is decided. Table 1 displays the Symbols and semantics used for experimental purpose.

**Table 1:** Symbols and semantics used for experimental purpose.

S. No.	Symbols	Semantics
1	$R$	Relative risk score
2	$\upmu$	Index sum
3		Leak impact factor
4		Third-party index
5		Corrosion index
6	$\vartheta$	Design index
7		In-correct operations
8		Product hazard
9		Leak volume
10		Dispersion
11		Receptors


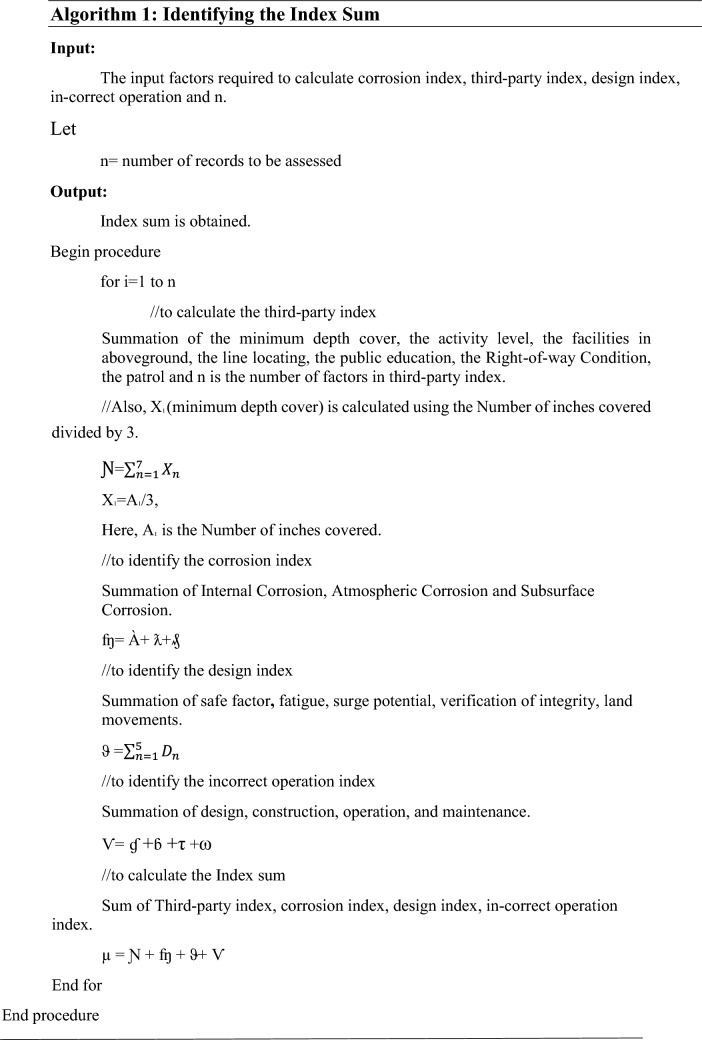
 
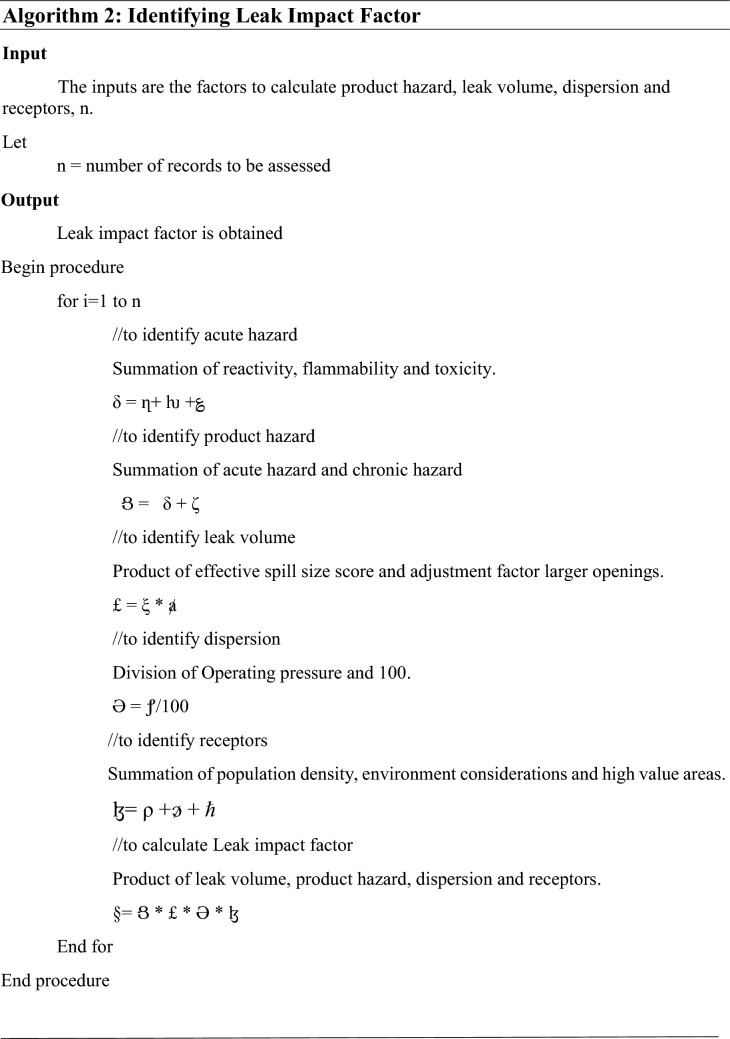


## Experimentation and analysis

### Experimental setup

This research concentrates on the quality and assesses the possible dangers of the pipeline. The assessment of the pipeline requires various factors, like environmental conditions, land movements, and other factors. The RRS methodology focuses on every factor that affects the pipeline and assesses the risk. Here, the relative risk score method is calculated by dividing the index sum by the leak impact factor. The dataset analysed for this research is published in Kaggle platform^43^ to collaborate with colab. Table 2 contains the risk level for the range of values of the relative risk score.

**Table 2:** Risk range and level.

Relative risk score range	Risk level
0–15	High
16–50	Medium
51–100	Low

The above-mentioned Table 2 is the range from which the risk level of pipeline is calculated.

### Results and discussion

The RRS is obtained by dividing the index sum by the leak impact factor. The index sum is calculated as the sum of the corrosion index, third-party index, design index, and in-correct operation index. The leak impact factor is calculated by the product of leak volume, product hazard, dispersion, and receptors. The risk level is marked based on the relative risk score range as depicted in Table 2. Table 3 depicts the statistical characteristics of the research data. Table 4 shows a sample of the calculated index sum, leak impact factor, relative risk score, and risk level.

**Table 3:** Statistical characteristics of the research data.

Minimum_Depth_Cover	Activity_Level	Above_Ground_Facilities	Line_Locating	Public_Education	Right_of_Way_Condition	Patrol	Atmospheric_Exposure	Atmospheric_Type	Atmospheric_Coating	Product_Corrosivity	Internal_Protection	Subsurface_Environment	Coating	Cathodic_Protection	Safety_Factor	Fatigue
45	8	4	2	2	3	8	4	1.2	1	3	4	5	15	10	28	8
38	16	10	6	1	5	0	5	2	3	10	10	5	15	15	7	15
40	13	1	2	2	1	12	2	0.8	2	1	3	15	10	15	35	14
43	5	3	4	2	3	8	1	0.5	2	1	5	5	15	10	14	8
48	1	1	2	4	1	12	0	1.6	2	1	2	15	10	15	35	9
55	4	2	2	1	0	15	3	2	1	3	3	5	15	10	14	10
52	2	3	5	4	3	8	2	0.8	2	1	5	5	15	10	28	11
60	1	4	2	4	2	10	2	0.8	1	3	5	5	15	15	7	12
57	5	3	4	2	1	12	3	2	2	1	4	15	10	10	21	13

Minimum_Depth_Cover	Surge_Potential	Integrity_verification	Land_Movement	Hazard_Identification	MAOP_Potential	Safety_Systems	Material_Selection	Checks	Inspection	Materials	Joining	Backfills	Handling	Coat	Procedures	Communication
45	5	5	5	4	10	3	− 2	− 2	5	1	1	1	1	2	7	3
38	5	10	10	4	12	6	− 2	− 2	4	0	2	2	0	2	7	3
40	0	15	5	4	5	10	− 2	− 2	8	1	0	0	0	1	7	3
43	10	5	0	0	5	1	− 2	− 2	9	2	1	2	2	1	7	3
48	5	15	5	4	10	6	− 2	− 2	4	1	2	1	2	0	7	3
55	5	5	10	4	5	10	− 2	− 2	5	1	0	2	1	2	7	3
52	10	10	5	4	10	− 2	− 2	− 2	6	2	2	1	2	1	7	3
60	0	5	0	0	10	6	− 2	− 2	8	2	2	0	2	0	7	3
57	0	10	5	4	5	3	− 2	− 2	9	2	2	0	1	1	7	3

Minimum_Depth_Cover	Drug_Testing	Safety_Programs	Survey/Maps/Records	Training	Mechanical_Error_Preventers	Documention	Schedule	Procedures	Acute_Hazard	Chronic_Hazard	Leak_Volume	Population_Density	Environmental_Issues	Environmental_Sensitivity	High_Value_Areas	Dispersion
45	2	2	5	10	1	2	3	10	4	10	0.18	10	10	0.8	3.5	0.76
38	2	2	5	10	2	2	3	10	3	8	0.2	9	10	0.6	3.5	0.55
40	2	2	5	10	4	2	3	10	7	5	0.2	4	10	0.5	5	0.54
43	2	2	5	10	4	0	0	0	5	7	0.19	7	8	0.3	2	0.62
48	2	2	5	10	4	2	3	10	8	5	0.19	8	10	0.7	4	0.61
55	2	2	5	10	2	2	3	10	1	2	0.18	4	10	0.5	5	0.46
52	2	2	5	10	1	2	3	10	10	8	0.19	2	5	0	2	0.45
60	2	2	5	10	1	0	0	0	12	3	0.2	1	5	0	2	0.71
57	2	2	5	10	2	2	3	10	11	8	0.18	7	10	0.5	3.5	0.66

**Table 4:** Index sum, leak impact factor, relative risk score and risk level.

S. No.	Index_sum	Leak_impact_factor	Relative_Risk_Score	Risk level
0	196.000000	18.29472	10.713474	High
1	208.200000	46.53936	4.473633	High
2	239.666667	27.95100	8.574529	High
3	233.133333	25.27200	9.224966	High
4	175.833333	24.45528	7.189995	High
5	229.600000	34.20209	6.713040	High
6	203.333333	4.84380	41.978061	Medium
7	222.133333	13.85100	16.037350	Medium
8	167.800000	17.04000	9.847418	High
9	209.000000	47.40120	4.409171	High
10	252.333333	7.65600	32.958899	Medium

**Figure 2:**
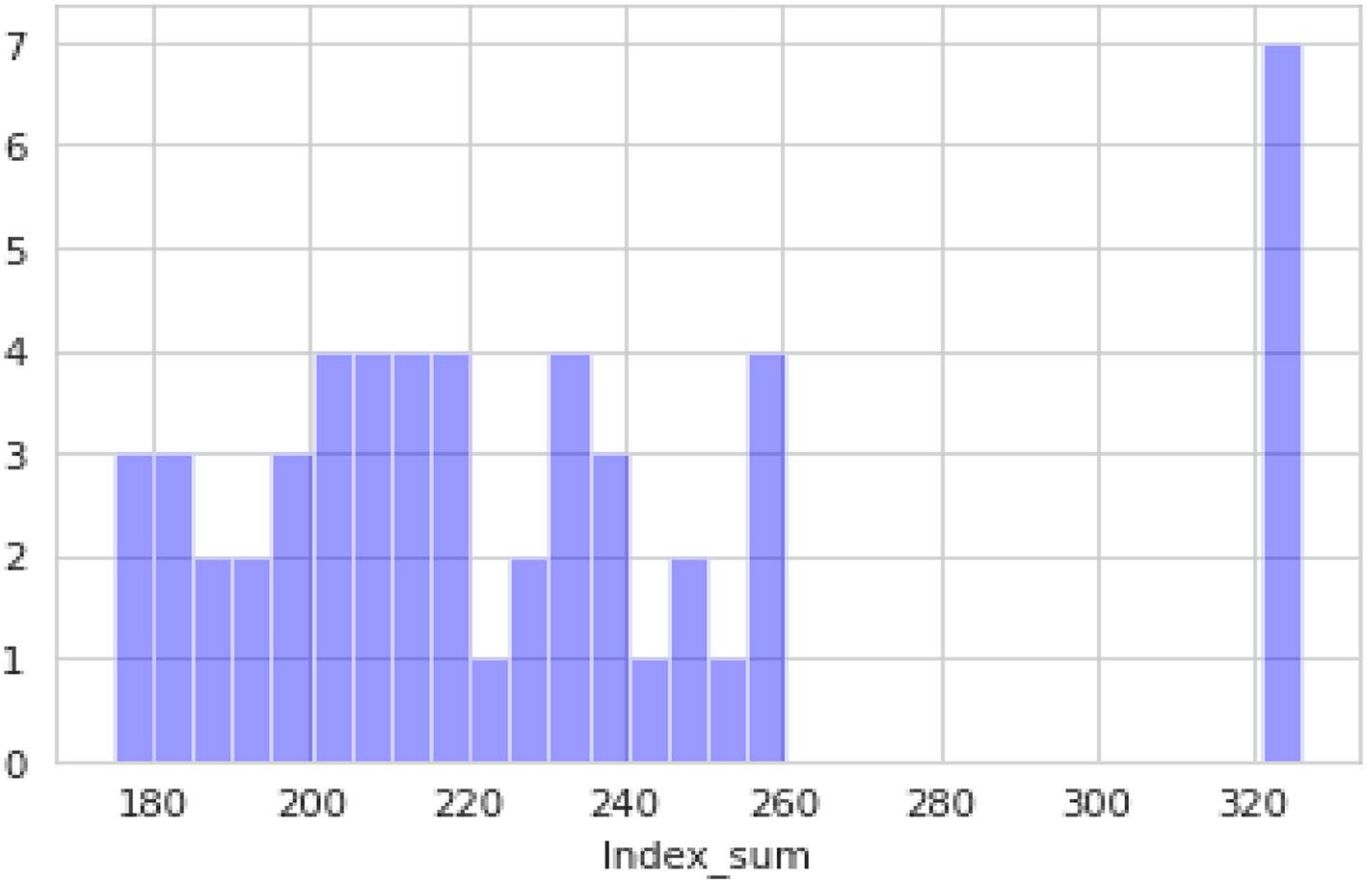
Index sum of pipelines.

Figure 2 depicts the range in which the values of the index sum lie. Here, the x-axis represents the values from the dataset, and the y-axis represents the ranges. The index sum values consist of corrosion index, third-party index, design index, and in-correct operation. All these four factors have several subfactors, in which the values are added and the final value is given as the index sum.

**Figure 3:**
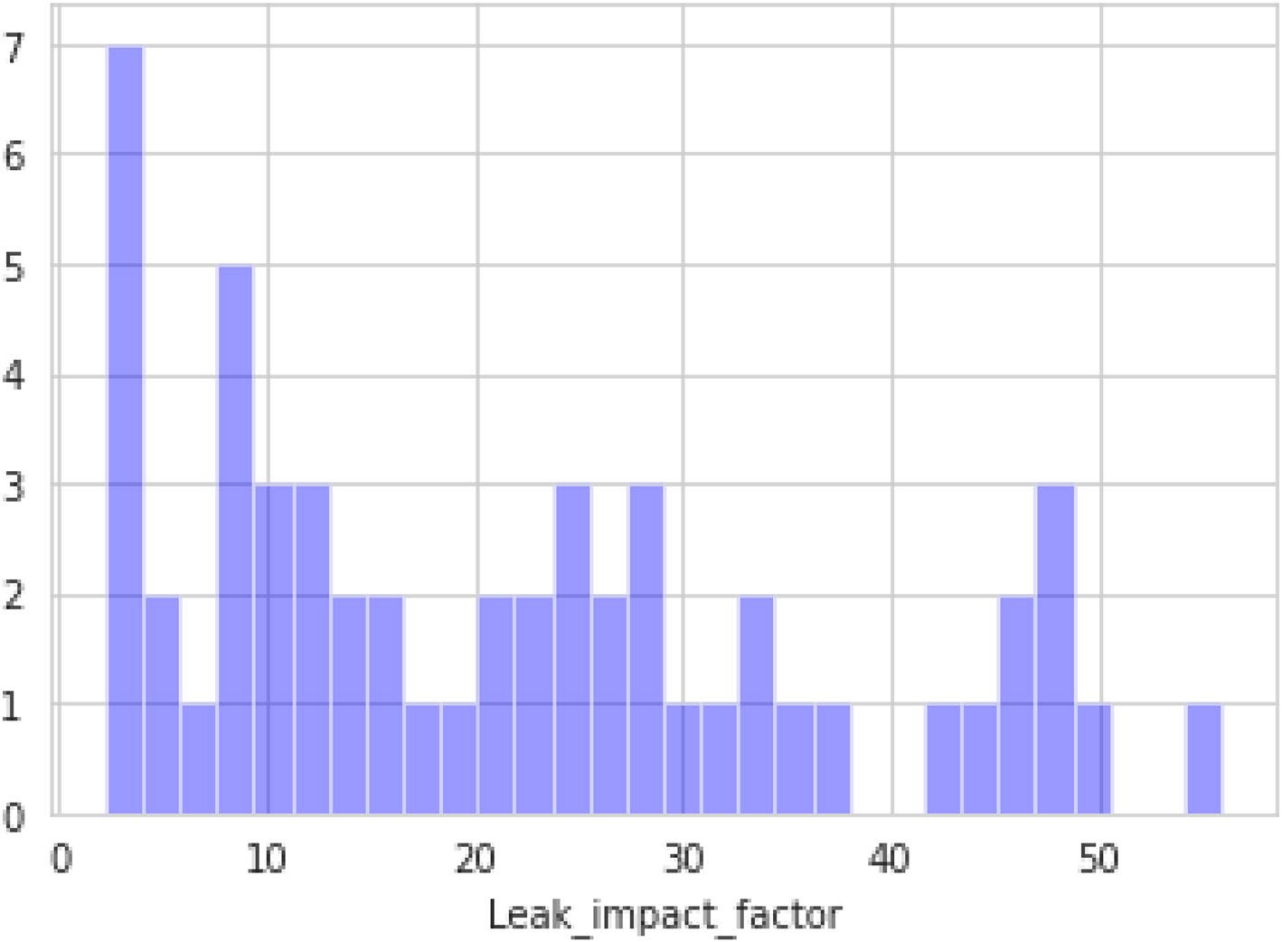
Leak impact factor of pipelines.

Figure 3 depicts the range in which the values of the leak impact factor lie. Here, the x-axis represents the values from the dataset, and the y-axis represents the ranges. The leak impact factor values consist of product hazards, leak volume, dispersion, and receptors. Each of these four factors has several subfactors, the values are multiplied and the final value given as the leak impact factor.

**Figure 4:**
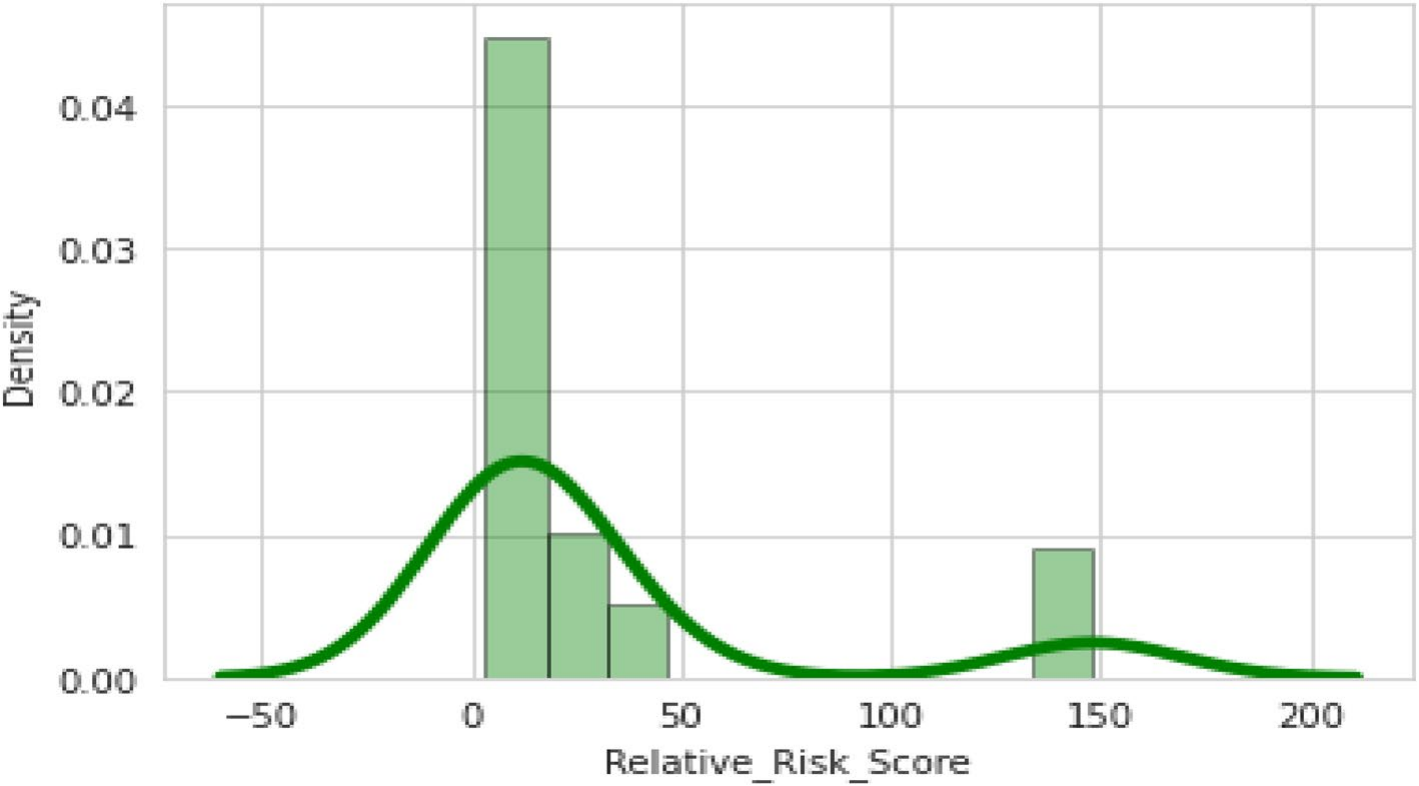
Relative Risk Score.

Figure 4 depicts the observations on a single attribute, which is univariate, and visualizes it through a histogram, i.e., only one observation. Here, the relative risk score is observed. The relative risk score is calculated by dividing the index sum by the leak impact factor.

**Figure 5:**
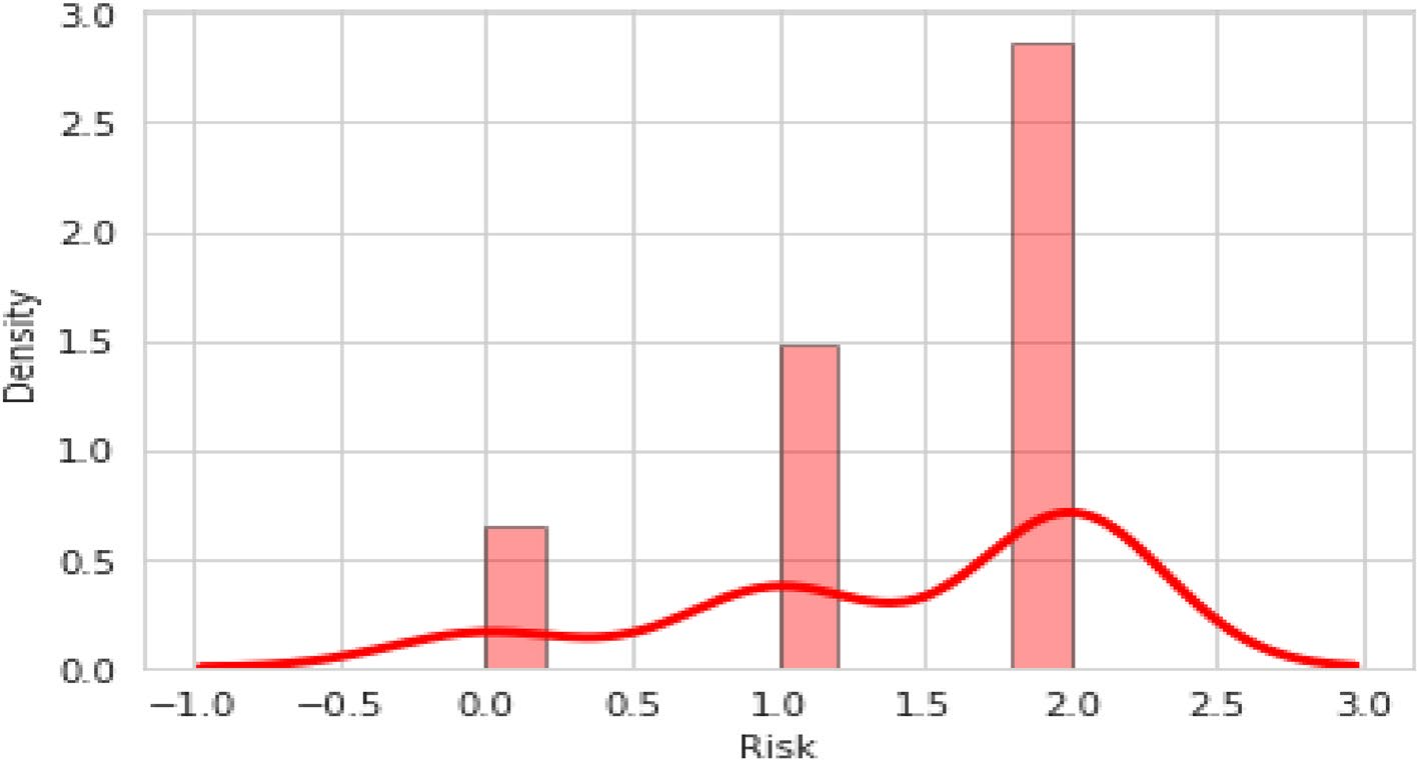
Risk of the pipeline.

Figure 5 also depicts the observations on a single attribute which is univariate and visualizes it through a histogram. Here, the risk of the pipeline is observed. From the relative risk score method, the risk of the pipeline is calculated as high, low, or medium based on the input values.

**Figure 6:**
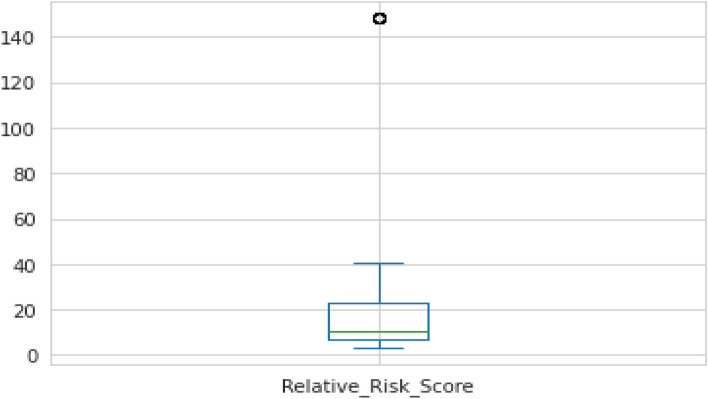
Relative risk score.

Figure 6 shows the relative risk score’s box plot. The main objective of the box plot is to find the minimum value, maximum value, and outliers. Outlier detection is the process of identifying an unknown observation in a given dataset.

**Figure 7:**
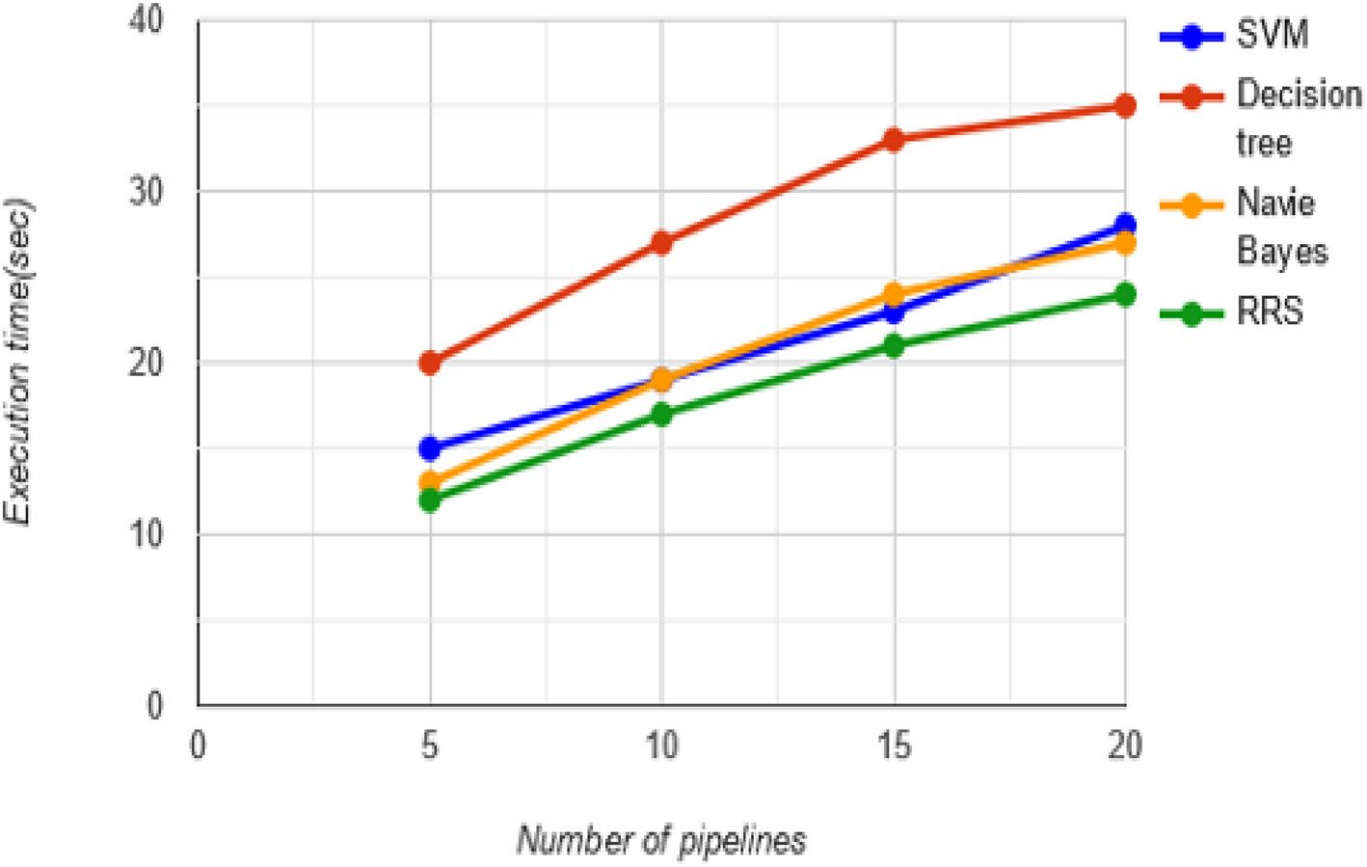
Performance comparison of RRS method with existing algorithms.

Figure 7 depicts the comparison of various methodologies, i.e., support vector machines, decision trees, and naive bayes algorithms, with the RRS methodology. These algorithms are compared based on the execution time. In this way the RRS method shows the better execution time (24 ms) while comparing the other algorithms. This comparison proves that the submitted methodology is better than the existing methodologies in terms of performance.

In Table 5 the performance metrics like accuracy, precision, recall and F1 score of RRS methodology are discussed.

**Table 5:** Performance metrics of the RRS method.

Accuracy	Precision	Recall	F1-score
97.5%	0.95	0.93	0.94

**Figure 8:**
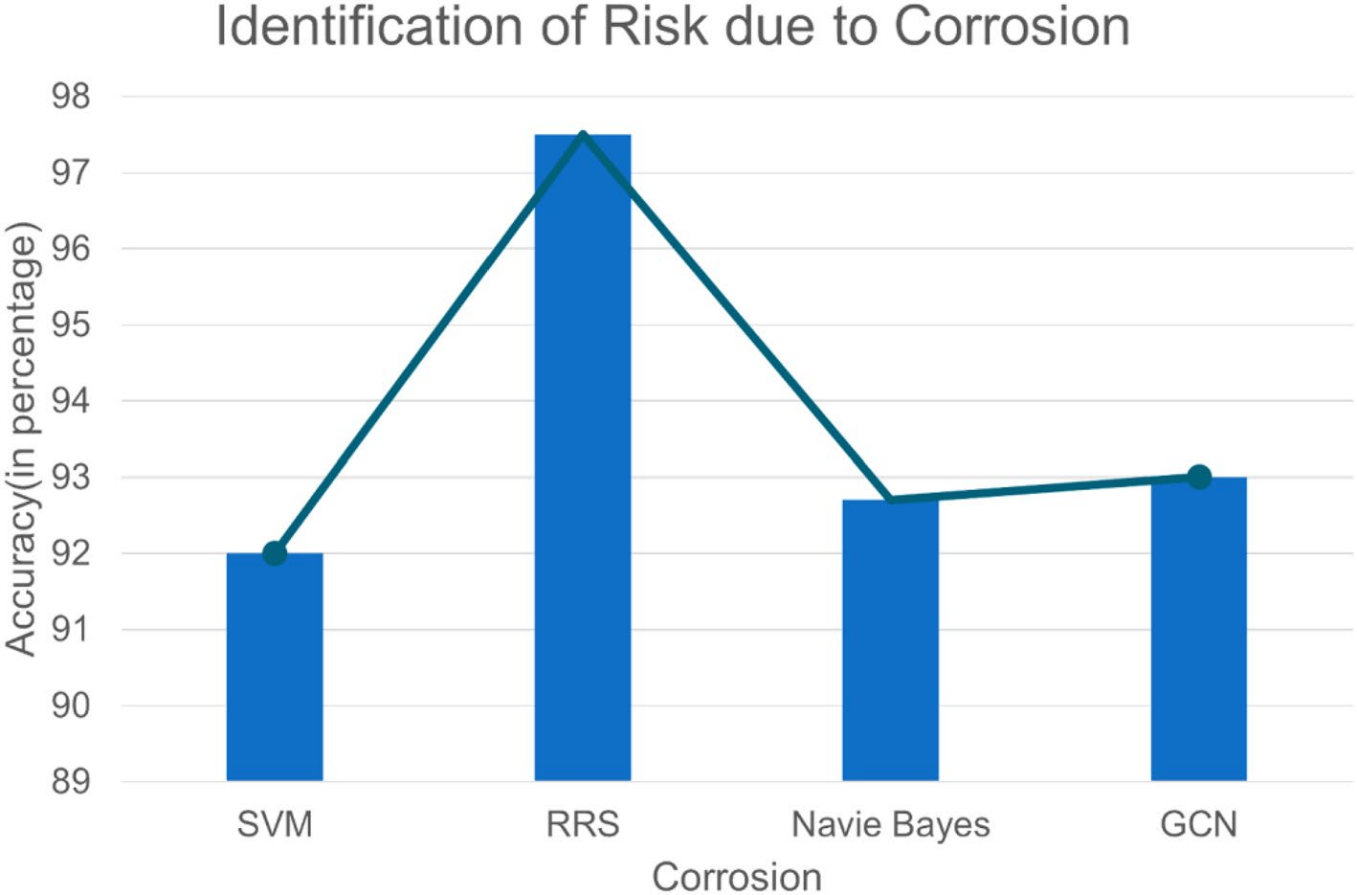
Identification of Risk due to corrosion.

The above Fig. 8 depicts the graph for identifying the risk due to corrosion. The x-axis represents the corrosion in different algorithms, and the y-axis represents the accuracy in percentage. The percentage of pipeline corrosion is compared here using various algorithms such as Naive Bayes, support vector machine (SVM), and graph convolutional network (GCN). The SVM shows 92%, the Naive Bayes algorithm shows 92.7%, the GCN algorithm shows 93%, and the RRS method shows the greatest accuracy percentage of 97.5%. When comparing these three algorithms, the Relative Risk Scoring method shows a high percentage of accuracy in predicting corrosion. Based on this analysis, the graph proves that the RRS method is the best method for calculating corrosion accuracy.

**Figure 9:**
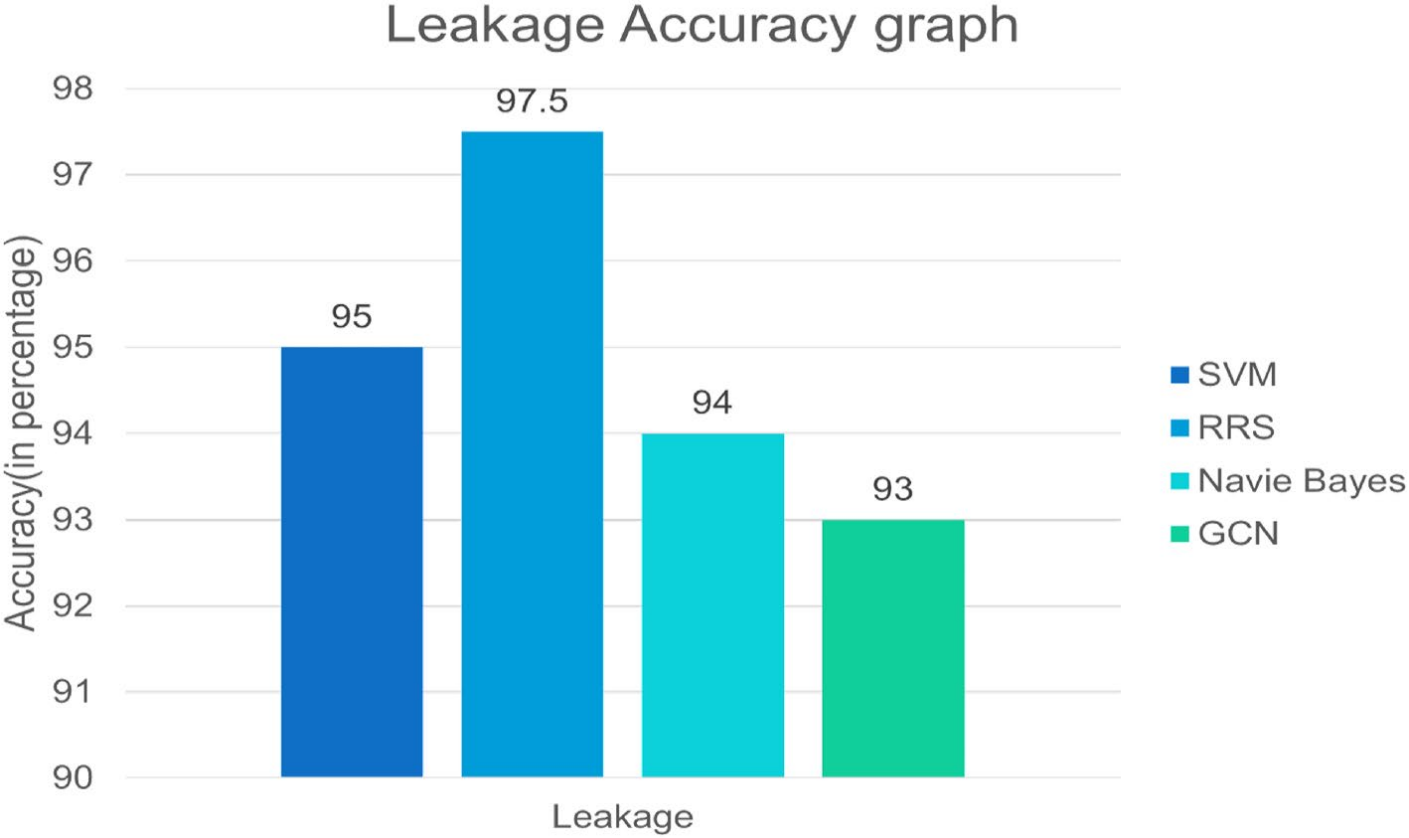
Identification of risk due to leakage.

The Fig. 9 depicts a graph for identifying the risk due to leakage. The x-axis represents the leakage in different algorithms, and the y-axis represents the accuracy in percentage. Support vector machine (SVM), the Naive Bayes algorithm, and the graph convolutional. The SVM shows 95%, Naive Bayes algorithm shows 94%, GCN algorithm shows 93%, and RRS method shows the greatest accuracy percentage of 97.5%. When these three algorithms are compared, the Relative Risk Scoring method has the highest percentage accuracy in detecting leakage. Based on this analysis, the graph shows that the RRS method is the most effective method for calculating leakage accuracy. From above Figs. 8 and 9, it is proven that SVM and GCN are individually better in the aspect of predicting leakage and corrosion but, RRS is better than them in both aspects.

The methodology was carefully designed and tested to ensure that it is reliable. The RRS methodology has achieved only 93% of recall which can be improved with further addition of experiments. The proposed methodology has several advantages, the risk of the pipeline is predicted using various parameters including corrosion, leakage and other damages so that the risk of the pipeline is not over calculated. Thus, the leakage, corrosion and other damages are not predicted separately. Hence, this method is more accurate to calculate the risk of the pipeline than other methodologies.

## Conclusion

In conclusion, pipelines serve as the backbone of the global transportation network for products such as oil, water, and gas. However, the safe and efficient operation of pipelines requires comprehensive risk assessment. Our study presents the RRS method, a novel approach to risk assessment that considers a wide range of parameters, including those often overlooked in existing methodologies. The RRS algorithm provides more accurate results for calculating leakage, corrosion, and classification, with accuracies of 96.5%, 94.7%, and 94.3%, respectively. Compared to the Decision Tree algorithm, the RRS method executes much faster, reducing the time and cost associated with risk assessment. Overall, the RRS method represents a significant advancement in pipeline risk assessment. Its ability to provide more accurate results, faster execution time, and comprehensive consideration of all parameters makes it a more reliable and efficient approach. The RRS method has the potential to improve the safety and efficiency of pipeline operation in the future, providing a safer, more cost-effective, and more sustainable approach to transporting products across the globe. Thus, we conclude that the RRS method is a reliable and efficient approach for risk assessment of pipelines.

## Data Availability

The datasets generated and/or analysed during the current study are available in the Kaggle^43^ repository, [https://www.kaggle.com/datasets/vanitham20bsr059/oilchemical-pipeline-dataset].
